# The In Vitro Effects of Pentamidine Isethionate on Coagulation and Fibrinolysis

**DOI:** 10.3390/molecules24112146

**Published:** 2019-06-06

**Authors:** Rami A. Al-Horani, Daytriona Clemons, Madhusoodanan Mottamal

**Affiliations:** 1 Division of Basic Pharmaceutical Sciences, College of Pharmacy, Xavier University of Louisiana, New Orleans, LA 70125, USA; dclemons@xula.edu; 2 Department of Chemistry, Xavier University of Louisiana, New Orleans, LA 70125, USA; mmottama@xula.edu

**Keywords:** pentamidine, factor Xa, plasmin, coagulation, fibrinolysis

## Abstract

Pentamidine is bis-oxybenzamidine-based antiprotozoal drug. The parenteral use of pentamidine appears to affect the processes of blood coagulation and/or fibrinolysis resulting in rare but potentially life-threatening blood clot formation. Pentamidine was also found to cause disseminated intravascular coagulation syndrome. To investigate the potential underlying molecular mechanism(s) of pentamidine’s effects on coagulation and fibrinolysis, we studied its effects on clotting times in normal and deficient human plasmas. Using normal plasma, pentamidine isethionate doubled the activated partial thromboplastin time at 27.5 µM, doubled the prothrombin time at 45.7 µM, and weakly doubled the thrombin time at 158.17 µM. Using plasmas deficient of factors VIIa, IXa, XIa, or XIIa, the concentrations to double the activated partial thromboplastin time were similar to that obtained using normal plasma. Pentamidine also inhibited plasmin-mediated clot lysis with half-maximal inhibitory concentration (IC_50_) value of ~3.6 μM. Chromogenic substrate hydrolysis assays indicated that pentamidine inhibits factor Xa and plasmin with IC_50_ values of 10.4 µM and 8.4 µM, respectively. Interestingly, it did not significantly inhibit thrombin, factor XIa, factor XIIIa, neutrophil elastase, or chymotrypsin at the highest concentrations tested. Michaelis-Menten kinetics and molecular modeling studies revealed that pentamidine inhibits factor Xa and plasmin in a competitive fashion. Overall, this study provides quantitative mechanistic insights into the in vitro effects of pentamidine isethionate on coagulation and fibrinolysis via the disruption of the proteolytic activity of factor Xa and plasmin.

## 1. Introduction

**Figure 1:**
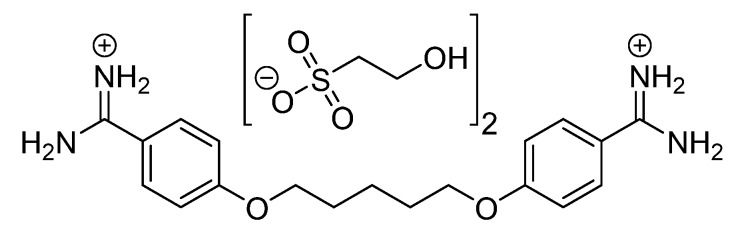
Chemical structure of pentamidine isethionate. Pentamidine is the active constituent and is chemically known as 4,4′-(pentane-1,5-diylbis(oxy))-dibenzimidamide.

Pentamidine isethionate (Figure 1) is an antimicrobial drug that is used parenterally or by inhalation for the treatment of African trypanosomiasis, babesiosis, and leishmaniasis. Given its strong basic characteristics, pentamidine does not significantly cross the blood brain barrier, and thus, it is only used for the early stage of African trypanosomiasis at which there is no central nervous system involvement [1,2,3]. It is also used in the treatment of *Pneumocystis jirovecii* pneumonia which is a major opportunistic infection in immune compromised patients including patients with acquired immunodeficiency disease [4].

Parenteral administration of pentamidine should be cautiously performed in patients with pre-existing bone marrow depression or blood dyscrasias. In fact, complete blood count should be performed before, periodically during, and after therapy. This is, in part, because pentamidine is associated with significant hematological toxicities including leukopenia (10%), thrombocytopenia (3%), anemia (1%), neutropenia, and pancytopenia [5]. Rare but potentially life-threatening blood clot formation has also been observed in less than 1% of treated patients. The development of disseminated intravascular coagulation following pentamidine administration was also reported [6].

Previous in vitro studies indicated that pentamidine isethionate dose-dependently inhibits platelet function. At concentrations greater than 8.4 µM, pentamidine completely inhibited platelet-aggregation induced by adenosine diphosphate, epinephrine, thrombin, and collagen as well as partially inhibited retention and clot retraction in platelet-rich plasmas. Likewise, pentamidine was reported to prolong clotting times at concentrations greater than 8.4 µM [7]. 

In this report, we investigated the potential in vitro molecular mechanisms of pentamidine isethionate effects on coagulation and fibrinolysis. We quantitatively examined the effect of this drug on the activated partial thromboplastin time (APTT) using normal human plasma as well as deficient plasmas of certain coagulation factors. Furthermore, we quantified the effect of the drug on prothrombin time (PT) and thrombin time (TT) in normal human plasmas. We also in vitro quantified the effect of pentamidine isethionate on plasmin-mediated clot lysis. The inhibitory potential of pentamidine isethionate towards coagulation proteins (human thrombin and factors Xa, XIa, and XIIIa), fibrinolysis protease (human plasmin), digestive proteases (bovine trypsin and chymotrypsin), and inflammatory protease (human neutrophil elastase) was also studied. As a result, we report that pentamidine isethionate doubled the APTT, PT, and TT at concentrations of 27.88, 45.70, and 158.17, respectively. It inhibited the plasmin-mediated clot lysis with IC_50_ value of 3.6 ± 1.5 µM and efficacy of 58.3 ± 8.9%. Pentamidine isethionate was found to be a moderately potent inhibitor of human factor Xa (FXa), a serine protease of the common coagulation pathway, and of human plasmin, a serine protease of fibrinolysis, with IC_50_ values of 10.4 ± 0.8 µM and 8.4 ± 0.8 µM, respectively. Michaelis-Menten kinetics and molecular modeling studies indicated that pentamidine inhibits the two enzymes competitively by targeting select amino acid residues in the enzymes’ active sites.

## 2. Materials and Methods

### 2.1. Chemicals, Reagents, Enzymes, and Substrates

Pentamidine isethionate was purchased from Millipore-Sigma (St. Louis, MO, USA). Reagents for clotting assays including thromboplastin-D, APTT reagent, and thrombin time reagent were all from Fisher Scientific (Pittsburgh, PA, USA). Chemicals used to prepare enzyme assay buffers were from Millipore-Sigma or Fisher Scientific. *N*,*N*–dimethylcasein, dansylcadaverine, and dithiothreitol were also from Millipore-Sigma. All types of plasmas were purchased from George King Bio-Medical, Inc. (Overland Park, KS, USA). Coagulation enzymes including thrombin, factor Xa (FXa), factor XIa (FXIa), and factor XIIIa (FXIIIa) were from Haematologic Technologies, Inc. (Essex Junction, VT, USA). Digestive enzymes including trypsin and chymotrypsin were from Millipore-Sigma. Neutrophil elastase was from Elastin Products Company (Owensville, MO, USA). Chromogenic substrates: Spectrozyme TH, Spectrozyme FXa, and Spectrozyme PL, were obtained from Biomedica-Diagnostics (Windsor, NS, Canada). FXIa chromogenic substrate (S-2366) and trypsin substrate (S-2222) were obtained from Diapharma (West Chester, OH, USA). Chromogenic substrate for chymotrypsin (*N*-Succinyl-Ala-Ala-Pro-Phe-*p*-nitroanilide) and that for neutrophil elastase (S-1384) were from Millipore-Sigma.

### 2.2. In vitro Effect on Clotting Times of Human Plasmas

Effect of pentamidine isethionate on clotting times of normal human plasma was evaluated in a standard one-stage assay using BBL Fibrosystem fibrometer (Becton-Dickinson, Sparles, MD, USA), as described previously [8,9]. For the PT assay, thromboplastin-D was reconstituted according to the manufacturer’s directions and warmed to 37 °C. A volume of 10 μL of variable concentrations of pentamidine isethionate was brought up to 100 μL with citrated human plasma, incubated for 30 sec at 37 °C followed by the addition of 200 μL of prewarmed thromboplastin-D, and the time to clotting was noted. For the APTT assay, 10 μL of variable concentrations of pentamidine isethionate was mixed with 90 μL of citrated human plasma and 100 μL of prewarmed APTT reagent (0.2% ellagic acid). After incubation for 4 min at 37 °C, clotting was initiated by adding 100 μL of prewarmed 25 mM CaCl_2_ and the time to clotting was noted. For the TT assay, 10 μL of variable concentrations of pentamidine isethionate was brought up to 200 μL with citrated human plasma, incubated for 3 min at 37 °C followed by the addition of 100 μL of prewarmed thrombin time reagent, and the time to clotting was noted. The data were fit to a quadratic trendline, which was used to determine the concentration of pentamidine isethionate necessary to double the clotting time. Clotting times in the absence of pentamidine isethionate were determined in a similar fashion using 10 μL of highly purified water and were found to be 14.9 ± 0.5 s for PT, 31.4 ± 0.5 s for APTT, and 9.6 ± 0.1 s for TT. To determine the in vitro effect on the APTT of deficient human plasmas, the APTT assay was repeated, using human plasmas deficient of factor VII (FVII), factor IX (FIX), FXI, or factor XII (FXII). Isethionic acid did not affect the APTT at the highest concentration tested of 33.3 mM. The positive control used in APTT and PT assays was rivaroxaban, a clinically used FXa inhibitor. Under the conditions reported, rivaroxaban doubled the APTT and PT at concentrations of 0.123 ± 0.003 µM and 0.193 ± 0.043 µM, respectively. 

### 2.3. In Vitro Effect on Fibrinolysis

The method followed here was similar to that reported earlier [10]. This in vitro experiment was conducted in 96-well platform in pH 7.4 buffer at 25 °C. The clot was generated by the addition of 80 µL of a solution of human thrombin (2.5 µg/mL) in 20 mM TrisHCl buffer containing 10 mM CaCl_2_ to 60 µL of a solution of fibrinogen (10 mg/mL) and FXIIIa (2 µg/mL) in 20 mM TrisHCl buffer containing 10 mM CaCl_2_. The resulting clot was stabilized for 15 min at 25 °C. The absorbance was then measured at 405 nm and was ~0.98 across all wells. Following, 5 µL of pentamidine isethionate (or water) (effective concentrations in the well were 0–1900 µM) was added to each well resulting in no significant change in the A405 of the corresponding wells. Finally, 5 µL of human plasmin (stock of 1.6 µM) in 20 mM TrisHCl buffer of pH 7.4 containing 2.5 mM CaCl_2_ and 100 mM NaCl was added to each well and the A405 of each well was recorded over more than 340 min at various time intervals. By semilog plotting the rate of clot lysis (fibrinolysis) over the time period of 5–100 min versus the corresponding concentrations of pentamidine isethionate using the logistic Equation (1), the IC_50_ of fibrinolysis inhibition and the inhibition efficacy were determined. In this equation, Y is the percentage ratio of clot lysis in the presence of pentamidine isethionate to that in its absence, Y_M_ and Y_0_ are the maximum and minimum possible values of the clot lysis (%), IC_50_ is the concentration of the test molecule inhibitor that results in 50% inhibition of clot lysis, and HS is the Hill slope.

(1) $$Y=Y_{0}+\frac{Y_{M}-Y_{0}}{1+10^{\left(log{\left[I\right]}_{0}-logIC_{50}\right)\left(HS\right)}}$$

### 2.4. Effect of Pentamidine Isethionate on Common Coagulation Pathway and Fibrinolysis Enzymes

Direct inhibition of human FXa and α-thrombin of the common coagulation pathway and human plasmin of the fibrinolysis pathway were measured using the corresponding chromogenic substrate hydrolysis assays at 37 °C and pH 7.4, as reported earlier using the 96-microplate platform [8,9,10,11,12]. In FXa assay, each well of a 96-well microplate generally contained 185 µL pH 7.4 buffer to which 5 µL pentamidine isethionate (or purified water) and 5 µL FXa (43.5 nM stock conc.) were sequentially added. After 10 min incubation, 5 µL Spectrozyme FXa (5 mM stock conc.) was rapidly added and the residual activity of FXa was measured from the initial rate of increase in absorbance at 405 nm. The relative residual enzyme activity at each concentration of pentamidine isethionate was calculated from the ratio of enzyme activity in the presence and absence of the test molecule. The concentration vs effect curve was constructed using logistic Equation (1) from which the potency (IC_50_) and efficacy (ΔY) of inhibition were also obtained. In Equation (1), Y is the percentage ratio of residual enzyme activity in the presence of pentamidine isethionate to that in its absence, Y_M_ and Y_0_ are the maximum and minimum possible values of the residual FXa activity (%), IC_50_ is the concentration of the test molecule that results in 50% inhibition of FXa activity.

For thrombin and plasmin, the assays were performed using substrates appropriate for each enzyme (Spectrozyme TH for thrombin and Spectrozyme PL for plasmin) under conditions closest to the physiological condition. The K_M_ of the substrate for its enzyme was used to identify the concentration of the substrate to be used for inhibition studies. For thrombin assay, each well of a 96-well microplate generally contained 185 µL pH 7.4 buffer to which 5 µL pentamidine isethionate (or reference) and 5 µL thrombin (240 nM stock conc.) were sequentially added. After 10 min incubation, 5 µL Spectrozyme TH (1.0 mM stock conc.) was rapidly added and the residual activity of thrombin was measured from the initial rate of increase in absorbance at 405 nm. For plasmin assay, each well of a 96-well microplate generally contained 85 µL pH 7.4 buffer to which 5 µL pentamidine isethionate (or purified water) and 5 µL plasmin (500 nM stock conc.) were sequentially added. After 10 min incubation, 5 µL Spectrozyme PL (1.0 mM stock conc.) was rapidly added and the residual activity of plasmin was measured from the initial rate of increase in absorbance at 405 nm. The potency and efficacy of plasmin inhibition by pentamidine isethionate were calculated as above. Buffer for assays was 20–50 mM Tris-HCl buffer, pH 7.4, containing 100–150 mM NaCl, 2.5 mM CaCl_2_, 0.1% PEG8000, and 0.02% Tween80. All assays were performed three times.

### 2.5. Effect of Pentamidine Isethionate on Related Enzymes

Direct inhibition of a panel of human serine proteases including the coagulation protease FXIa, the inflammation protease neutrophil elastase, and the digestive proteases trypsin and chymotrypsin by pentamidine isethionate was also evaluated using the corresponding chromogenic substrate hydrolysis assays in a 96-well microplate format adapted from the literature [8,9,10,11,12,13]. Direct inhibition of the human coagulation transglutaminase FXIIIa was also studied using a fluorescence-based transglutamination assay as reported in the literature [14,15].

For the FXIa assay, each well of a 96-well microplate generally contained 85 µL pH 7.4 buffer to which 5 µL pentamidine isethionate and 5 µL plasma FXIa (15.3 nM stock conc.) were sequentially added. After 10 min incubation, 5 µL FXIa substrate (6.9 mM stock concentration) was rapidly added and the residual FXIa activity was measured from the initial rate of increase in absorbance at 405 nm. Assays were performed in similar fashion with trypsin and chymotrypsin. Stock concentrations used were 18.9 µg/mL (trypsin) and 22.7 mM (S-2222) for bovine trypsin assay (final volume was 200 µL) and 2 µg/mL (chymotrypsin) and 16.2 mM (*N*-Succinyl-Ala-Ala-Pro-Phe-*p*-nitroanilide) for bovine chymotrypsin assay (final volume was 200 µL). The buffer used for FXIa, trypsin, chymotrypsin assays was 50 mM Tris-HCl buffer, pH 7.4, containing 150 mM NaCl, 2.5 mM CaCl_2_, 0.1% PEG8000, and 0.02% Tween80. Neutrophil elastase inhibition assay was performed in a 96-well plate with a final volume of 100 µl with 0.125 M 4-(2-hydroxyethyl)-1-piperazineethanesulfonic acid (HEPES) buffer, pH 7.4 containing 0.125% Triton-X 100, as reported earlier [13]. To each well containing 88 µL of the buffer was added 4 µL neutrophil elastase (2 µM stock conc.) and 5 µL pentamidine isethionate. After incubation, 3 µL neutrophil elastase substrate (S-1384; 25 mM stock conc.) was rapidly added and the residual enzyme activity measured from the initial rate of increase in the absorbance at 405 nm.

For the FXIIIa assay, 1 μL of pentamidine isethionate was diluted with 87 μL of pH 7.4 buffer (50 mM Tris-HCl, 1 mM CaCl_2_, 100 mM NaCl, and 2 mg/mL *N*,*N*–dimethylcasein) and 5 μL dithiothreitol (20 mM) at 37 °C, followed by the addition of 2 μL human FXIIIa (0.3 μM) and incubation for 10 min. The activity of FXIIIa was evaluated by the addition of 5 μL dansylcadaverine (2 mM) and measuring the initial rate of increase in fluorescence emission (λ_Ex._ = 360 nm and λ_Em._ = 490 nm). In all assays, the dose dependence of the fractional residual enzyme activity, if any, was analyzed using Equation (1) above to obtain the apparent concentration of the test molecule required to reduce the enzyme activity to 50% of its initial value [14,15]. All assays were performed three times.

### 2.6. Michaelis-Menten Kinetics for Chromogenic Substrates Hydrolysis by FXa and Plasmin in the Presence of Pentamidine Isethionate

The initial rate of Spectrozyme FXa hydrolysis by human FXa (87 nM) or that of Spectrozyme PL hydrolysis by human plasmin (120 nM) was monitored from the linear increase in absorbance at 405 nm corresponding to less than 10% consumption of the substrate. The initial rate was measured as a function of various concentrations of the substrate (0–500 µM) in the presence of fixed concentration of pentamidine isethionate (0–100 μM) in 20–50 mM TrisHCl buffer, pH 7.4, containing 100–150 mM NaCl, 2.5 mM CaCl_2_, 0.1% PEG8000, and 0.02% Tween80 at 37 °C. The data were fitted by Michaelis-Menten Equation (2) to determine K_M_ (substrate affinity to the enzyme) and V_MAX_ (maximum velocity of enzyme reaction).

(2) $$V_{i}=\frac{V_{MAX}\times \left[S\right]}{K_{M}+\left[S\right]}$$

### 2.7. Molecular Modeling of Pentamidine Binding to the Active Site of Human FXa and Plasmin

Molecular docking studies were performed to evaluate the binding mode of pentamidine to the active sites of human FXa and plasmin using Schrodinger’s (Suite 2015-3) Glide 6.8 program [16]. We used the coordinates of FXa from the co-crystal structure of FXa-DX-9065a complex (PDB ID:1FAX) [17]. Likewise, the coordinates of plasmin were retrieved from the ternary complex of microplasmin-staphylokinase-microplasmin which also includes the modified tripeptide GluGlyArg-chloromethyl-ketone (PDB ID:1BUI) [18]. The structures of FXa and plasmin were prepared by removing crystallographic water molecules beyond 5 Å from the ligand and adding hydrogen atoms consistent with the physiologic pH of 7.0 using Maestro 10.3 of Schrodinger Suite. In the case of plasmin, covalent bonds of the inhibitor to the amino acid residues His57 and Ser195 were also deleted. Subsequently, the protein structures were energy minimized with a root-mean-square deviation (RMSD) cutoff value of 0.3 Å for all heavy atoms. Initial coordinates for pentamidine were extracted from ZINC database [19] and the molecule was prepared by energy minimization using the Schrodinger Suite. The co-crystallized ligand region and the area surrounding it was specified as the ligand binding site, and the receptor grids for each target protein was generated using the OPLS3 forcefield [20]. The grid center was set to be the centroid of the co-crystal ligand, with a cubic grid box of 10 Å on each side. No constraints were used in any of the receptor grid generations. The docking calculations were done using the default parameters under the standard precision mode. All the poses were subjected to post-docking minimization. The best-docked structure based on the docking score was selected for subsequent analysis.

## 3. Results

### 3.1. Pentamidine Affects the Clotting Times of Human Plasma and the Effect Is Independent of Factors VIIa, IXa, XIa, and XIIa

**Figure 2:**
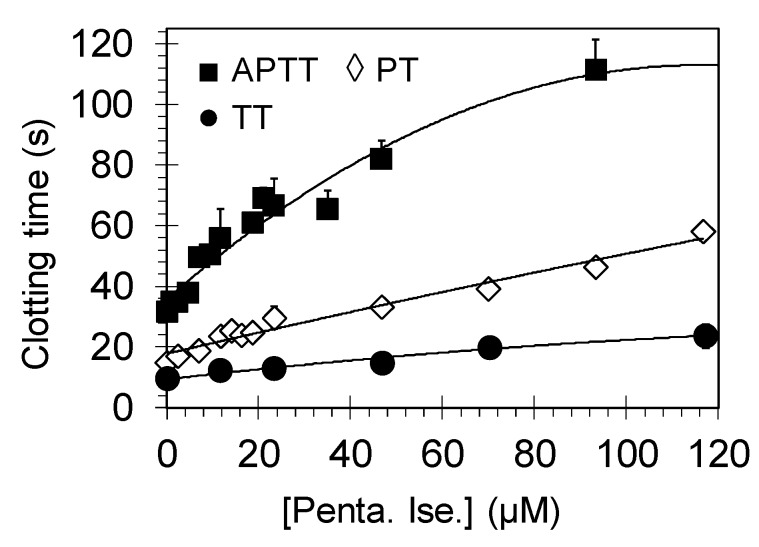
Effect of pentamidine isethionate on the clotting times of APTT, PT, and TT in normal human plasma. Shown is the prolongation of clotting time as a function of pentamidine isethionate concentration (0–120 µM) in either APTT assay (■), PT assay (◊), or TT assay (●). Solid lines are trend lines from which the concentration necessary to double the clotting time was deduced. Given also are the standard error bars (±1 SE). Penta. Ise. = Pentamidine isethionate.

Plasma clotting assays including APTT, PT, and TT are routinely used to assess the potential effect of molecules on the coagulation process in an in vitro setting. The APTT assay typically measures the effect of molecules on the intrinsic pathway of coagulation which involves FXIIa, FXIa, and FIXa, in addition to the effect on the common coagulation pathway, particularly on thrombin and FXa. Likewise, the PT assay evaluates the effect of molecules on the extrinsic pathway of coagulation involving FVIIa, in addition to the effect on the common pathway. The TT screens the effect on the formation of fibrin from fibrinogen after the addition of known amounts of thrombin to the plasma sample [21]. The concentrations of pentamidine required to double APTT, PT, and TT were measured, as described earlier [8,9]. Figure 2 shows the variations in the three clotting times in the presence of varying concentrations of pentamidine isethionate. A two-fold increase in the APTT required 27.88 ± 2.99 µM from pentamidine isethionate in human plasma containing all clotting factors. Interestingly, the two-fold increase in the APTT using plasmas deficient of FVII, FIX, FXI, or FXII was achieved with similar concentrations (Table 1). This suggests that the effect of pentamidine isethionate on the APTT is independent of FVIIa, FIXa, FXIa, or FXIIa. Likewise, a two-fold increase in the PT and TT required 45.70 ± 0.85 µM and 158.17 ± 30.87 µM of pentamidine isethionate, respectively. Under the reported conditions, isethionic acid did not affect the APTT in normal human plasma at the highest concentration tested of 33.3 mM. Rivaroxaban, a clinically used FXa inhibitor, doubled the APTT and PT at concentrations of 0.123 ± 0.003 µM and 0.193 ± 0.043 µM, respectively, which is consistent with reported values [22,23,24]. These results indicate that pentamidine more effectively prolongs the APTT and PT in in vitro setting. Furthermore, the dual effect on APTT and PT and the lack of statistically significant changes in the concentration required to double the APTT in plasmas deficient of clotting factors of intrinsic and extrinsic pathways indicate that the effect of pentamidine can be attributed to effect(s) on proteins in the common coagulation pathway, namely thrombin and/or FXa. Nevertheless, the two-fold increase in TT required a relatively high concentration of >150 µM in normal plasma which subsequently suggests that the effect of pentamidine on clotting times may be primarily attributed to FXa inhibition. It is worth to mention here that the positive control rivaroxaban has no effect on TT [22,23,24].

### 3.2. Pentamidine Dose-Dependently Inhibits Plasmin-Mediated Clot Lysis

**Figure 3:**
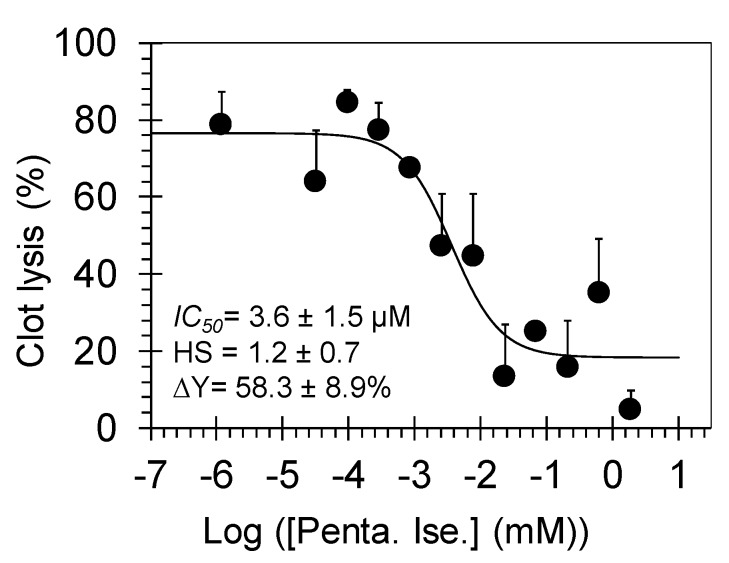
The dose-dependent inhibition of plasmin-mediated clot lysis by pentamidine isethionate. The effect was determined by measuring the ultraviolet (UV) absorbance of each well containing different concentration of pentamidine at 405 nm over a time period of 340 min. Solid line represents the sigmoidal dose–response fits (Equation (1)) for the data to obtain the values of IC_50_, HS, and ΔY (%). Given also are the standard error bars (±1 SE).

A key experiment to evaluate the effect of pentamidine isethionate on the clot lysis is the in vitro plasmin-mediated clot fibrinolysis assay in a 96-well platform [10]. A thrombin-induced fibrin-rich clot was first generated by inducing fibrinogenolysis using human thrombin and FXIIIa in 20 mM TrisHCl, pH 7.4, containing 10 mM CaCl_2_ at 25 °C. The resulting clot was allowed to stabilize for 15 min, following which, pentamidine isethionate (or water) at desired concentrations was added and allowed to equilibrate. Finally, clot lysis was initiated by the addition of human plasmin and was monitored by measuring absorbance at 405 nm as a function of time. Plasmin-mediated hydrolysis of the fibrin-rich clot resulted in a decrease in the relative A405 over time. Under the conditions studied, the clot was completely lysed in the absence of pentamidine isethionate in 340 min. Yet, a significant inhibition of clot lysis was detected in the presence of pentamidine isethionate in a dose–dependent manner. Plotting the rate of clot lysis (i.e., fibrinolysis) over 5–100 min versus the corresponding concentrations of pentamidine isethionate shows a semilog sigmoidal relationship, which could be analyzed using dose-response Equation (1) to calculate the IC_50_ of fibrinolysis inhibition. An apparent IC_50_ of 3.6 ± 1.5 μM and efficacy of 58.3 ± 8.9% (Figure 3) was calculated using this non-linear regressional analysis. 

### 3.3. Pentamidine Isethionate Selectively Inhibits Human FXa and Plasmin in the Chromogenic Substrate Hydrolysis Assays

**Figure 4:**
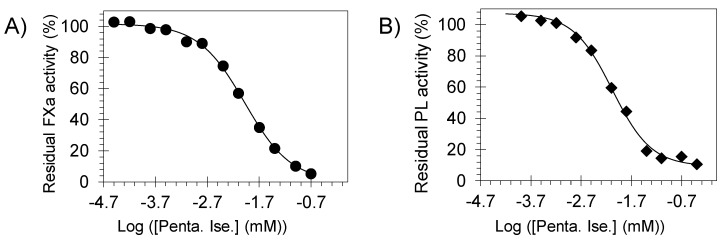
Direct inhibition of human FXa (●, **A**) and human plasmin (♦, **B**) by pentamidine isethionate (0–400 µM). Solid lines represent sigmoidal dose−response fit (Equation (1)) for the data to obtain the values of IC_50_, HS, and ΔY (%).

To identify the molecular targets which represent the foundation for pentamidine isethionate effects on clot formation and lysis, we tested the inhibitory potential of this molecule towards human coagulation and fibrinolysis proteins including thrombin, FXa, FXIa, FXIIIa, and plasmin using 96-well platform and under the physiological conditions of pH 7.4 and 37 °C (Table 2). In chromogenic substrate hydrolysis assays [8,10,11,12], pentamidine dose-dependently inhibited FXa and plasmin with IC_50_ values of 10.4 ± 0.8 µM (Figure 4A) and 8.4 ± 0.8 µM (Figure 4B), respectively. Yet, it did not significantly inhibit thrombin or FXIa at the highest concentration tested of 100 µM (inhibition was <40% at 100 µM). In fluorescence-based, bi-substrate transglutamination assay [14,15], pentamidine isethionate did not inhibit human FXIIIa at the highest concentration tested of 50 µM. In fact, pentamidine did not inhibit the prototypical guinea pig transglutaminase at concentrations as high as 500 µM. Likewise, pentamidine isethionate also did not inhibit other serine proteases of bovine chymotrypsin and human neutrophil elastase at the highest concentrations tested of 1750 µM and 100 µM, respectively. Yet, it inhibited bovine trypsin with IC_50_ values of 4.9 ± 0.8 µM under similar physiological conditions (Table 2). 

**Figure 5:**
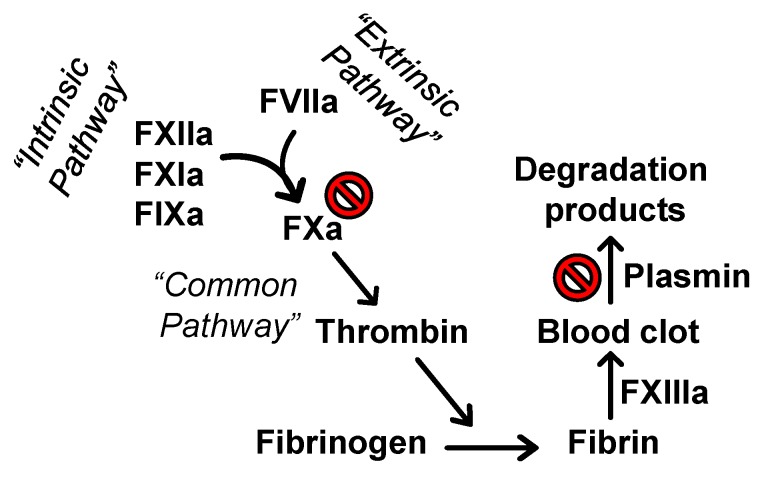
Representation of the coagulation (clot formation) and fibrinolysis (clot lysis) processes. The coagulation proceeds extrinsically (FVIIa) and/or intrinsically (FXIIa, FXIa, and FIXa). The two pathways further promote coagulation through the common coagulation pathway involving thrombin and FXa. Thrombin activates fibrinogen to fibrin which gets cross-linked by the action of FXIIIa. The resulting insoluble mesh of fibrin entraps activated platelets as well as red blood cells to form the blood clot. Following the clot formation, the clot undergoes plasmin-mediated lysis. The in vitro effect of pentamidine isethionate on coagulation and fibrinolysis is, in part, due to its inhibitory effects on FXa and plasmin, respectively.

Overall, these results suggest that pentamidine is a relatively selective inhibitor of few serine proteases and that its effect on clot formation and lysis is because of its inhibition of FXa and plasmin, respectively, as depicted in Figure 5. 

### 3.4. Michaelis-Menten Kinetics of FXa and Plasmin Inhibition by Pentamidine Isethionate

**Figure 6:**
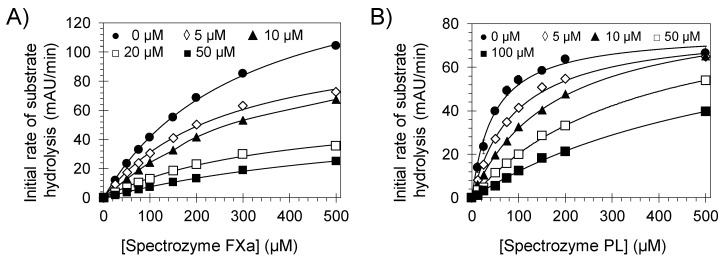
Michaelis-Menten kinetics of human FXa (**A**) and human plasmin (**B**) in the presence of different concentrations of pentamidine isethionate (0–100 µM). The initial rate of hydrolysis at various substrate concentrations was measured spectrophotometrically in pH 7.4 buffer at 37 °C. Solid lines represent the nonlinear regressional fits for the data using Equation (2) to yield K_M_ and V_MAX_.

To understand the basis for pentamidine’s inhibitory potential towards human FXa, the kinetics of Spectrozyme FXa hydrolysis by FXa was measured at pH 7.4 and 37 °C in the presence of five different concentrations (0–50 µM) of the substrate. As expected, the initial velocity varied in a hyperbolic manner with increasing concentration of the substrate at all concentrations of pentamidine (Figure 6A). The K_M_ for Spectrozyme FXa in the absence of pentamidine was found to be 305.0 ± 11.8 µM (Table 3), which increased ~2.3-fold upon increasing the inhibitor’s concentration to 50 μM (690.2 ± 63.1 µM). At the same time, the V_MAX_ decreased ~2.8-fold from 169.4 ± 3.6 mAU/min to 60.3 ± 3.8 mAU/min for the same range of pentamidine concentration. These results indicate that pentamidine is a mixed inhibitor of FXa. In general, a mixed inhibitor may bind to both the free enzyme and the enzyme-substrate complex but has greater affinity for one form over the other. In this case, the affinity of Spectrozyme FXa to the enzyme decreases with increasing the concentration of pentamidine suggesting that the inhibitor may prefer to bind to the free enzyme, and thus, the inhibition kinetics mimic the kinetics of a competitive inhibitor.

Likewise, to understand the basis for pentamidine’s inhibitory potential towards human plasmin, the kinetics of Spectrozyme PL hydrolysis by plasmin was measured at pH 7.4 and 37 °C in the presence of five different concentrations (0–100 µM) of the substrate. As with FXa, the initial velocity varied in a hyperbolic manner with increasing concentration of Spectrozyme PL at all concentrations of pentamidine (Figure 6B). The K_M_ for Spectrozyme PL in the absence of pentamidine was found to be 46.5 ± 5.1 µM (Table 3), which increased ~13.9-fold upon increasing the inhibitor’s concentration to 100 μM (645.5 ± 68.17 µM). At the same time, the V_MAX_ remained essentially constant for the same range of pentamidine concentration. These results indicate that pentamidine competitively inhibits the catalytic function of plasmin. Overall, pentamidine isethionate inhibits FXa and plasmin by targeting their active sites.

### 3.5. Molecular Modeling Studies of Pentamidine Interaction with FXa and Plasmin

**Figure 7:**
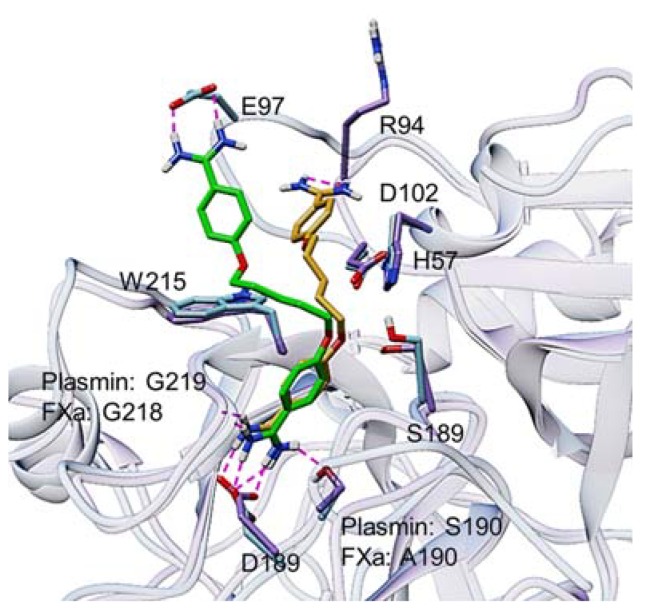
Glide-based docking and scoring study of pentamidine binding to human FXa (PDB ID: 1FAX) (sky blue protein and green inhibitor) and plasmin (PDB ID:1BUI) (purple protein and gold inhibitor). The key interactions are shown as dash lines and pentamidine is shown as sticks. For the two proteins, one of the inhibitor’s protonated amidine groups is engaged with D189 residue.

To better understand the interactions between pentamidine and the two enzymes, FXa and plasmin, at atomic levels, we performed molecular modeling studies as described in the Materials and Methods. In the two exercises involving the two trypsin-like serine proteases, the best-docked structures indicate that one protonated amidino group of pentamidine forms a characteristic salt bridge to the side chain carboxylate group of D189 residue and a hydrogen bond to the carbonyl group of G219 residue in the S1 subsite of the active site of each enzyme (Figure 7). However, the other protonated amidino group appears to engage via another salt-bridge interaction with the carboxylate group of the side chain of E97 residue in FXa or via hydrogen bonds with the backbone carbonyl oxygen of R94 residue in plasmin. Overall, these results are in line with the fact that pentamidine is competitive inhibitor of FXa and plasmin and it disrupts their catalytic activities by targeting their active sites.

## 4. Discussion and Significance

Considering the coagulation process, the results demonstrate that pentamidine significantly and dose-dependently doubles the clotting times APTT and PT at concentrations lower than 50 µM under in vitro conditions, and it doubles the TT, albeit at concentrations higher than 150 µM. This suggests that pentamidine’s in vitro effect on the coagulation process stems, in part, from targeting proteins belonging to the common coagulation pathway, particularly FXa which was inhibited dose-dependently and competitively by pentamidine with IC_50_ value of ~10 µM and efficacy of ~100%. Importantly, the effect of pentamidine at this concentration level appears to be specific due to the lack of effect on thrombin, FVIIa, FIXa, FXIa, FXIIa, or FXIIIa as indicated by studies involving deficient plasmas as well as the corresponding enzyme inhibition assays. Considering the fibrinolysis process, pentamidine significantly and dose-dependently inhibits the fibrinolysis process (clot lysis) in vitro settings by targeting plasmin. Pentamidine’s inhibition potency and efficacy towards plasmin is substrate-dependent phenomena. Using the physiological substrate of plasmin i.e., cross-linked plasma clot, pentamidine inhibits plasmin with IC_50_ of ~3.6 µM and efficacy of ~58%. Yet, in the chromogenic substrate hydrolysis assay using the chromogenic tripeptide Spectrozyme PL, pentamidine competitively inhibits plasmin with IC_50_ value of ~8.4 µM and efficacy of ~100%. 

Accordingly, the above findings along with the previously reported effects on platelet aggregation [7], may together account for the effect of pentamidine on coagulation and fibrinolysis in vitro. Important to mention here is that the relevance of these results to clinical practice is unknown. Generally, the means of plasma C_MAX_ of pentamidine isethionate is ≤1µM [25,26,27] which suggests that pentamidine isethionate effects on coagulation and fibrinolysis may not be experienced by most of the patients. Yet, it should be noted that the likelihood of these adverse effects may potentially increase in patients having compromised renal or liver functions due to the involvement of these two organs in the metabolism and elimination of pentamidine and its metabolites [28].

Lastly, results reported here can be exploited to devise reversal strategies for pentamidine’s hematological toxicities. The results may also inspire the design of new anti-trypanosomiasis and anti-leishmaniasis drugs that lack potential effects on coagulation and/or fibrinolysis. This is particularly important considering that the other antiprotozoal drug that is used to treat the early stage African trypanosomiasis i.e., suramin also affects the coagulation process via inhibiting the thrombin-induced fibrinogen clotting [29]. Furthermore, although it inhibits trypsin, the specificity of pentamidine continues to be significant given the lack of significant effect on a wide range of coagulation enzymes, neutrophil elastase, and chymotrypsin. Therefore, the pentamidine chemical scaffold can be exploited as a platform to design new effective anticoagulants by targeting human FXa or effective antifibrinolytics by targeting human plasmin for parenteral use in hospital settings. 

## Figures and Tables

**Table 1:** The effect of pentamidine isethionate on the clotting times (APTT, PT, and TT) of different human plasmas. ^a)^

Type of Human Plasma	[Pentamidine Isethionate] (µm) to Double Clotting Time
**APTT assay**	**APTT_EC2×_**
Normal	27.88 ^b)^ ± 2.99 ^c)^
Deficient of FVII	26.71 ± 0.50
Deficient of FIX	26.32 ± 3.52
Deficient of FXI	29.48 ± 3.88
Deficient of FXII	27.05 ± 6.05
**PT assay**	**PT_EC2×_**
Normal	45.70 ± 0.85
**TT assay**	**TT_EC2×_**
Normal	158.17 ± 30.87

Note: ^a)^ Prolongation of clotting times as a function of concentration of pentamidine isethionate in APTT, PT, and TT assays, ^b)^ The effective concentrations of pentamidine isethionate required to double the clotting times when coagulation triggered by the intrinsic pathway (APTT_EC2×_), the extrinsic pathway (PT_EC2×_), and thrombin (TT_EC2×_). In each case, the concentration is the average of three trials, ^c)^ Errors represent ± 1 S.E.

**Table 2:** The inhibition profile of pentamidine isethionate towards several enzymes of relevance to coagulation, inflammation, and digestion. ^a)^

Enzyme	IC_50_ (µM)	HS	∆Y (%)
**Thrombin**	>100 ^b)^	NA ^c)^	NA
**FXa**	10.4 ± 0.8 ^d)^	0.97 ± 0.07	101.6 ± 2.9
**FXIa**	>100	NA	NA
**FXIIIa**	>50	NA	NA
**Plasmin**	8.4 ± 0.8	1.1 ± 0.1	98.0 ± 3.3
**Neutrophil Elastase**	>100	NA	NA
**Trypsin**	4.9 ± 0.8	1.0 ± 0.2	116.8 ± 7.4
**Chymotrypsin**	>1750	NA	NA

Notes^: a)^ The IC_50_, HS, and ∆Y values were obtained following non-linear regression analysis of direct inhibition of the enzyme. Inhibition was monitored by spectrophotometric measurement of residual proteases activity (see Materials and Methods), ^b)^ No inhibition was observed up to concentrations as high as 100 µM (thrombin, FXIa, and neutrophil elastase), 50 µM (FXIIIa), or 1750 µM (chymotrypsin), ^c)^ Not applicable, ^d)^ Errors represent ± 1 S.E.

**Table 3:** Hydrolysis of the chromogenic substrate Spectrozyme FXa by human FXa and Spectrozyme PL by human plasmin in the presence of pentamidine isethionate. ^a)^

**Inhibitor**	**Spectrozyme FXa**	
**Pentamidine** **Isethionate (µM)**	**K_M_ (µM)**	**V_MAX_ (mAU/min)**
0	305.0 ± 11.8 ^b)^	169.4 ± 3.6
5	266.8 ± 21.2	114.3 ± 4.7
10	410.3 ± 18.2	124.2 ± 3.3
20	364.4 ± 36.6	63.1 ± 3.6
50	690.2± 63.1	60.3 ± 3.8
**Inhibitor**	**Spectrozyme PL**	
**Pentamidine** **Isethionate (µM)**	**K_M_ (µM)**	**V_MAX_ (mAU/min)**
0	46.5 ± 5.1	76.2 ± 2.5
10	92.9 ± 6.2	78.7 ± 2.0
20	176.1 ± 4.3	88.4 ± 1.0
50	358.9 ± 21.3	92.9 ± 3.2
100	645.5 ± 68.17	90.9 ± 6.5

Note: ^a)^ K_M_ and V_MAX_ values of the chromogenic substrate hydrolysis by the corresponding enzymes (human FXa or plasmin) were measured as described under (Materials and Methods). mAU indicates milliabsorbance units, ^b)^ Error represents ± 1 S.E.
